# Bis(2,3,5,6-tetra-2-pyridylpyrazine-κ^3^ *N* ^2^,*N* ^1^,*N* ^6^)iron(II) bis­(dicyanamidate) 4.5-hydrate

**DOI:** 10.1107/S1600536810003363

**Published:** 2010-02-03

**Authors:** L. Callejo, N. De la Pinta, G. Madariaga, M.L. Fidalgo, R. Cortés

**Affiliations:** a Departamento de Química Inorgánica, Facultad de Ciencia y Tecnología, Universidad del País Vasco, Apdo. 644, E-48080 Bilbao, Spain; b Departamento de Física de la Materia Condensada, Facultad de Ciencia y Tecnología, Universidad del País Vasco, Apdo. 644, E-48080 Bilbao, Spain; c Departamento de Química Inorgánica, Facultad de Farmacia, Universidad del País Vasco, Apdo. 450, E-01080 Vitoria, Spain

## Abstract

In the title compound, [Fe(C_24_H_16_N_6_)_2_][N(CN)_2_]_2_·4.5H_2_O, the central iron(II) ion is hexa­coordinated by six N atoms of two tridentate 2,3,5,6-tetra-2-pyridylpyrazine (tppz) ligands. Two dicyanamide anions [dca or N(CN)_2_^−^] act as counter-ions, and 4.5 water mol­ecules act as solvation agents. The structure contains isolated cationic iron(II)–tppz complexes and the final neutrality is obtained with the two dicyanamide anions. One of the dicyanamide anions and a water mol­ecule are disordered with an occupancy ratio of 0.614 (8):0.386 (8). O—H⋯O, O—H⋯N and C—H⋯O hydrogen bonds involving dca, water and tppz mol­ecules are observed.

## Related literature

For related structures including [*M*(II)(tppz)_2_]^2+^ cations, see: Ruminski & Kiplinger (1990 ▶); Arana *et al.* (1992 ▶); Allis *et al.* (2004 ▶); Burkholder & Zubieta (2004 ▶); Lainé *et al.* (1995 ▶). For the application of a [Co(II)(tppz)_2_]^2+^ complex as a homogeneous catalyst, see: Königstein & Bauer (1994 ▶, 1997 ▶). For dicyanamido (dca) anions, see: He *et al.* (2002 ▶). Some H-atom positions were calculated using *HYDROGEN* (Nardelli, 1999 ▶).
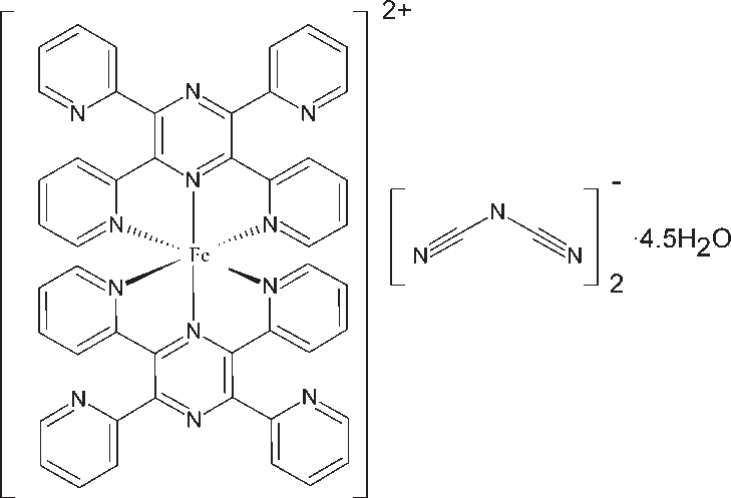


## Experimental

### 

#### Crystal data

[Fe(C_24_H_16_N_6_)_2_](C_2_N_3_)_2_·4.5H_2_O
*M* *_r_* = 1045.88
Monoclinic, 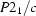

*a* = 13.9216 (7) Å
*b* = 18.9271 (9) Å
*c* = 19.1425 (9) Å
β = 97.017 (4)°
*V* = 5006.2 (4) Å^3^
*Z* = 4
Mo *K*α radiation
μ = 0.37 mm^−1^
*T* = 293 K
0.1 × 0.1 × 0.01 mm

#### Data collection

Oxford Diffraction Xcalibur 2 diffractometer
51234 measured reflections
15912 independent reflections
8433 reflections with *I* > 2σ(*I*)
*R*_int_ = 0.051

#### Refinement

*R*[*F* ^2^ > 2σ(*F* ^2^)] = 0.063
*wR*(*F* ^2^) = 0.168
*S* = 1.02
15912 reflections
779 parameters
135 restraints
H atoms treated by a mixture of independent and constrained refinement
Δρ_max_ = 0.86 e Å^−3^
Δρ_min_ = −0.34 e Å^−3^

### 

Data collection: *CrysAlis CCD* (Oxford Diffraction, 2007 ▶); cell refinement: *CrysAlis RED* (Oxford Diffraction, 2007 ▶); data reduction: *CrysAlis RED*; program(s) used to solve structure: *SHELXS86* (Sheldrick, 2008 ▶); program(s) used to refine structure: *SHELXL97* (Sheldrick, 2008 ▶); molecular graphics: *Mercury* (Macrae *et al.*, 2006 ▶); software used to prepare material for publication: *WinGX* (Farrugia, 1999 ▶).

## Supplementary Material

Crystal structure: contains datablocks I, global. DOI: 10.1107/S1600536810003363/zl2260sup1.cif

Structure factors: contains datablocks I. DOI: 10.1107/S1600536810003363/zl2260Isup2.hkl

Additional supplementary materials:  crystallographic information; 3D view; checkCIF report

## Figures and Tables

**Table 1:** Hydrogen-bond geometry (Å, °)

*D*—H⋯*A*	*D*—H	H⋯*A*	*D*⋯*A*	*D*—H⋯*A*
O1*W*—H11*W*⋯N13^i^	0.84 (2)	2.17 (3)	2.862 (5)	139 (3)
O1*W*—H12*W*⋯N12^i^	0.86 (2)	1.98 (2)	2.830 (4)	173 (3)
O2*W*—H21*W*⋯N15	0.85 (3)	2.16 (4)	2.903 (6)	146 (3)
O2*W*—H22*W*⋯O1*W*	0.86 (3)	1.96 (3)	2.785 (4)	160 (4)
O3*W*—H31*W*⋯N10	0.86 (11)	2.32 (13)	2.909 (10)	126 (11)
O3*W*—H32*W*⋯O5*WA*^ii^	0.86 (12)	2.29 (15)	2.87 (2)	126 (15)
O3*W*—H32*W*⋯O4*W*	0.86 (12)	2.11 (18)	2.46 (3)	104 (13)
O3*W*—H32*W*⋯O4*W*^ii^	0.86 (12)	2.41 (12)	3.24 (3)	162 (15)
O4*W*—H41*W*⋯O5*WA*	0.85 (7)	1.95 (9)	2.72 (4)	153 (17)
O5*WA*—H52*A*⋯N16*A*	0.85 (4)	2.19 (4)	2.92 (2)	143 (7)
O5*WB*—H51*B*⋯N6	0.85 (11)	2.52 (12)	3.188 (8)	136 (13)
O5*WB*—H52*B*⋯N16*B*	0.86 (8)	2.22 (7)	3.057 (18)	164 (10)
C8—H8⋯O4*W*^iii^	0.93	2.45	3.349 (16)	162

Symmetry codes: (i) 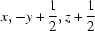; (ii) 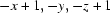; (iii) 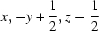.

**Fig. 1.**
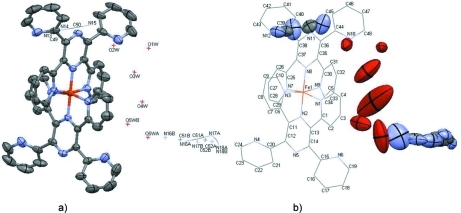
Asymmetric unit of [Fe(C24H16N6)2](N(CN)2)2.4.5H2O showing the most relevant labels. H atoms excluded for clarity. Ellipsoids drawn at 50% probability level.

**Fig. 2.**
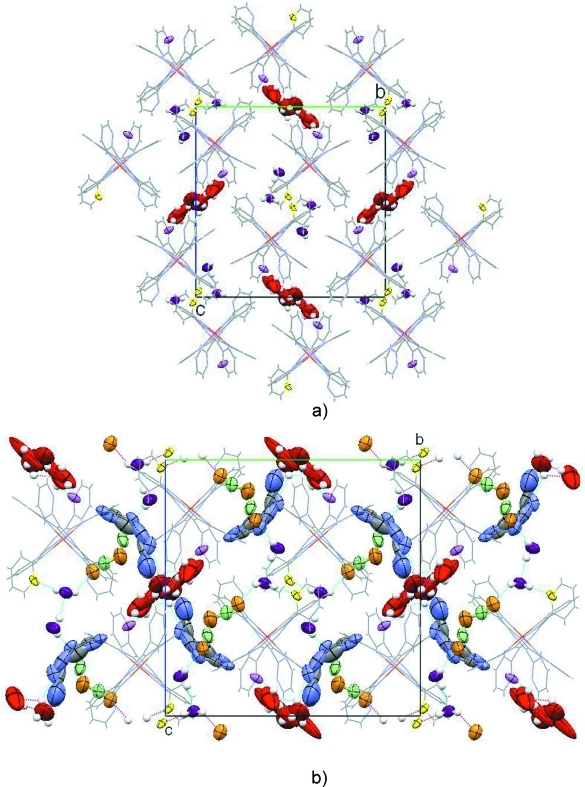
Projection of [Fe(C24H16N6)2](N(CN)2)2.4.5H2O packing along the a axis. In a) the voids occupied by solvents are shown. In b) are drawn the dca-O—O-dca chains. Infinite ordered dca chains are in green-orange colours. N10 and N12 atoms appear coloured in violet and yellow respectively. OW1 and OW2 in purple.

**Fig. 3.**
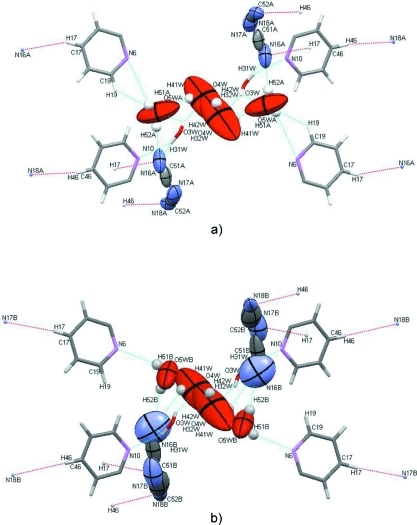
The two refined configurations for the disordered dca molecules. Occupancies are 0.386 (8) and 0.614 (8) for a) and b) respectively. Significant H-bonds are indicated.

**Fig. 4.**
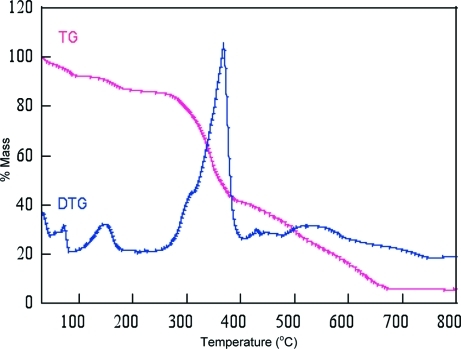
T G and DTG curves of [Fe(C24H16N6)2](N(CN)2)2.4.5H2O. The first stage [30–100] °C can be assigned to a loss of mass equivalent to 4.5 water molecules. Successive stages would correspond to the loss of 1 dca molecule [100–230] °C, 1 dca and 1 tppz molecules [236–395] °C and 1 molecule of tppz [398, 676] °C, respectively.

**Fig. 5.**
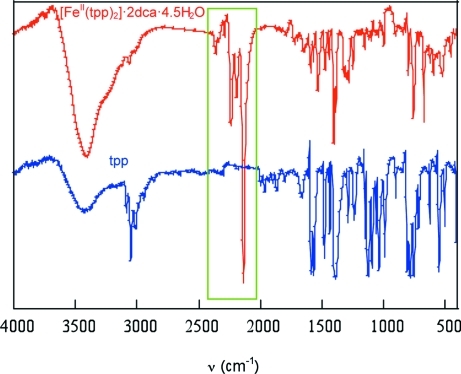
IR spectra of ttpz and [Fe(C24H16N6)2](N(CN)2)2.4.5H2O. The marked region include characteristic dca bands peaked at 2357 cm-1, 2288 cm-1, 2234 cm-1, 2190 cm-1 and 2136 cm-1 respectively.

## supplementary crystallographic information

### Comment

There is a noticeable number of investigations of transition metal complexes containing the ligand tppz, as these materials may possess desirable magnetic properties. The ligand tppz allows to synthesize extended conjugated systems where the (usually antiferro-) magnetic interactions are propagated through the tppz ligand itself. The combination of divalent cations of the second half of the transition series with the ligand tppz gives coordination cations of the type [M^II^(tppz)_2_]^2+^, where the terminal nitrogen atoms of one extreme of the tppz ligand are directed towards the metallic atoms and the corresponding N atoms of the other extreme remain uncoordinated (Lainé *et al.*, 1995). tppz ligands are part of other Fe^II^ (Allis *et al.*, 2004, Arana *et al.*, 1992, Ruminski & Kiplinger, 1990), Zn^II^ (Burkholder & Zubieta, 2004) and Co^II^ (Königstein & Bauer, 1994, Königstein & Bauer, 1997) compounds.

We here report the crystal structure of a new compound, [Fe(C_24_H_16_N_6_)_2_](N(CN)_2_)_2_.4.5H_2_O, which is made up of Fe^II^ cations coordinated to six nitrogen atoms of two tridentate tppz ligands. These monomeric entities reach neutrality with two dicyanamido (dca) anions (He *et al.*, 2002) and 4.5 molecules of water act as solvent agents (see Fig. 1). The coordinated nitrogen atoms of each tppz are in the same plane. The Fe^2+^cation has a distorted octahedral environment, in which the bonds to the two pyrazine nitrogen atoms (Fe1—N2 and Fe1—N8) are significantly shorter than the bonds to the pyridyl nitrogen atoms (Fe1—N1, Fe1—N3, Fe1—N7 and Fe1—N9). The N—Fe1—N angles involving the atoms of the equatorial plane with respect to the short axis differ remarkably from the ideal octahedral values, the Fe1 atom does not deviate significantly [-0.0035 (3) Å] from the average plane.

The individual pyridyl rings are planar (maximum average displacement with respect to the plane of the ring, 0.026 Å), while the two pyrazine rings are significantly puckered. Nitrogen atoms of the non-coordinated pyridyl rings of the two tppz ligands point to the ligand metalated side instead of the free nitrogen atom of the pyrazine ring. These structural features of the coordinated tppz ligand would account for its tendency to adopt bis-chelation in this type of complexes. Weak π-π interactions appear to occur due to overlap between pyridyl rings in the range 3.693 (2) Å to 3.9109 (13) Å, corresponding to the distances between the centroids of the rings labelled by N6 and N10^iv ^(iv= 1 + *x*,*y*,*z*) and N7 and N7^v ^(v= 1 - *x*,-*y*,-*z*), respectively.

The crystal packing of the bulky building block units leaves cavities of approximately 250 Å^3 ^where water molecules and dca anions are located (Fig. 2). The low density of the material reflects this open structure. One of the two crystallographically independent dca molecules is well defined. These ordered dca molecules, connected by water, form infinite chains aligned in the average along the c axis interconnecting the tppz ligands. The remainder of the dca anions and the water solvate molecules are disordered. They occupy large voids and contribute to link the tppz ligands by way of the N10 atoms (see Fig. 3). The second dicyanamide anions and a water molecule show a highly correlated disorder. Each of the disordered dca moieties (A and B) is bonded to the corresponding OW5 atom. These assemblies establish H bonds to O3W and the tppz ligands through N6 and C8 atoms. Additional plausible H bonds [generated by *Mercury* (Macrae *et al.*, 2006) but not tabulated owing to large D···A or H···A distances] involving the disordered O5W-dca groups appear in Fig. 3.

### Experimental

Crystals of [Fe(C_24_H_16_N_6_)_2_](N(CN)_2_)_2_.4.5H_2_O were prepared by mixing an acetonitrile solution (10 ml) of FeCl_2_.4H_2_O (99.4 mg, 0.50 mmol) and another acetonitrile solution (10 ml) of 2,3,5,6-tetra-2-pyridylpirazine (97.1 mg, 0.25 mmol). After vigorous stirring of about 30 minutes at a temperature of 303 K, an aqua/acetonitrile (50%) solution (10 ml) of sodium dicyanamide (111.3 mg, 1.25 mmol) was added. The resultant solution was stirred at 313 K for 25 minutes and at 298 K for the following two days. Then the orange precipitate that did form was filtered off. After evaporation of the mother liquor, dark purple prismatic crystals suitable for X-ray diffraction measurements formed. These crystals were found to be stable to X-ray exposure. The thermogravimetric analysis (Fig. 4) shows water loss at 373 K (7.8%Δm; theor.: 7.8%Δm) equivalent to 4.5 water molecules in agreement with the chemical analysis based on C (61.3%; theor.: 59.7%), N (24.7%; theor.: 24.1%) and H (4.0%; theor.: 3.6%). The IR spectrum (Figure 5) shows clearly the characteristic IR bands of dicyanamide in the range [2000, 2500] cm^-1^ (see the supporting material for details).

### Refinement

Structure solution by direct methods in the space group *P*2_1_/*c*, followed by refinement, based on F^2^, of atomic coordinates and anisotropic displacement parameters, was performed using the programs *SHELXS86* and *SHELXL97* (Sheldrick, 2008) successively. H atoms bonded to C atoms were found in successive difference Fourier maps and refined using a riding model, with C—H = 0.93 Å and with *U*_iso_(H) = 1.2U_eq_(C). C46 and C47 were restrained to have equal anisotropic displacements components along the direction of their bond. H atoms of O1W (see Fig. 1 for labelling) were located in a difference Fourier map whereas H atoms bonded to O2W, O3W, O4W and O5W were calculated with the program *HYDROGEN* (Nardelli, 1999). Water molecules were tightly restrained to the geometry used in *HYDROGEN* [O—H=0.85 (1) Å, H—O—H=107 (3)°] with *U*_iso_(H) = 1.5U_eq_(O). One of the dicyanamide groups, showing symptoms of disorder, was split in two positions and refined forcing its flatness with equal anisotropic displacement parameters for atoms closer than 1.7 Å and subject to a rigid bond restraint. Interatomic distances of the dca groups were assumed to be equal with a standard deviation of 0.02 Å. Also the distances O5W—N16 and H52—N16 were forced to be equal (within 0.02 Å) to those involving O1W—N13, O2W—N15 and H11W—N13 and H21W—N15 respectively. O4W appeared to be highly disordered around the inversion centre at (1/2,1/2,0) and was split in two equiprobable positions, the anisotropic refinement of its displacement parameters was restrained to approximate an isotropic behaviour. The water molecule O3W follows the statistical positions of OW4. Antibumping restrains of 2.20 (5) Å were applied to the closest distances H32W-(H51A, H52A) and H32W-(H41W, H42W). O5WA and O5WB are two mutually exclusive positions that were refined along with the two sets of atomic sites assigned to the disordered dicyanamide molecule. The refined site occupancy reaches a value of 0.386 (8) for the A positions. The highest residual electron density is 0.02 Å from atom Fe1 and the deepest hole is 0.62 Å from atom O4W.

### Crystal data

**Table:**

[Fe(C_24_H_16_N_6_)_2_](C_2_N_3_)_2_·4.5H_2_O	*F*(000) = 2164
*M**_r_* = 1045.88	*D*_x_ = 1.388 Mg m^−^^3^*D*_m_ = 1.39 (2) Mg m^−^^3^*D*_m_ measured by flotation
Monoclinic, *P*2_1_/*c*	Mo *K*α radiation, λ = 0.71073 Å
Hall symbol: -P 2ybc	Cell parameters from 8676 reflections
*a* = 13.9216 (7) Å	θ = 3.2–31.9°
*b* = 18.9271 (9) Å	µ = 0.37 mm^−^^1^
*c* = 19.1425 (9) Å	*T* = 293 K
β = 97.017 (4)°	Prism, black
*V* = 5006.2 (4) Å^3^	0.1 × 0.1 × 0.01 mm
*Z* = 4	

### Data collection

**Table:**

Oxford Diffraction Xcalibur 2 diffractometer	8433 reflections with *I* > 2σ(*I*)
Radiation source: sealed X-ray tube	*R*_int_ = 0.051
graphite	θ_max_ = 31.9°, θ_min_ = 3.2°
Detector resolution: 16.7 pixels mm^-1^	*h* = −20→16
656 images at 0.7^o^ stepwise rotation in ω and 90^o^ phi, 30 sec./frame scans	*k* = −28→26
51234 measured reflections	*l* = −27→27
15912 independent reflections	

### Refinement

**Table:**

Refinement on *F*^2^	Primary atom site location: structure-invariant direct methods
Least-squares matrix: full	Secondary atom site location: difference Fourier map
*R*[*F*^2^ > 2σ(*F*^2^)] = 0.063	Hydrogen site location: inferred from neighbouring sites
*wR*(*F*^2^) = 0.168	H atoms treated by a mixture of independent and constrained refinement
*S* = 1.02	*w* = 1/[σ^2^(*F*_o_^2^) + (0.0745*P*)^2^] where *P* = (*F*_o_^2^ + 2*F*_c_^2^)/3
15912 reflections	(Δ/σ)_max_ = 0.013
779 parameters	Δρ_max_ = 0.86 e Å^−^^3^
135 restraints	Δρ_min_ = −0.34 e Å^−^^3^
0 constraints	

### Special details

**Table:**

Geometry. All s.u.'s (except the s.u. in the dihedral angle between two l.s. planes) are estimated using the full covariance matrix. The cell s.u.'s are taken into account individually in the estimation of s.u.'s in distances, angles and torsion angles; correlations between s.u.'s in cell parameters are only used when they are defined by crystal symmetry. An approximate (isotropic) treatment of cell s.u.'s is used for estimating s.u.'s involving l.s. planes.
Refinement. Refinement of *F*^2^ against ALL reflections. The weighted *R*-factor *wR* and goodness of fit *S* are based on *F*^2^, conventional *R*-factors *R* are based on *F*, with *F* set to zero for negative *F*^2^. The threshold expression of *F*^2^ > σ(*F*^2^) is used only for calculating *R*-factors(gt) *etc*. and is not relevant to the choice of reflections for refinement. *R*-factors based on *F*^2^ are statistically about twice as large as those based on *F*, and *R*- factors based on ALL data will be even larger.

### Fractional atomic coordinates and isotropic or equivalent isotropic displacement parameters (Å^2^)

**Table:**

	*x*	*y*	*z*	*U*_iso_*/*U*_eq_	Occ. (<1)
Fe1	0.48936 (2)	0.100147 (16)	0.190355 (16)	0.03246 (11)	
N1	0.53441 (13)	0.03122 (10)	0.26346 (10)	0.0381 (4)	
N2	0.62308 (12)	0.11349 (9)	0.19270 (9)	0.0316 (4)	
N3	0.48732 (13)	0.17373 (9)	0.11775 (10)	0.0350 (4)	
N4	0.75663 (17)	0.19528 (12)	0.02476 (13)	0.0587 (6)	
N5	0.81757 (13)	0.13007 (10)	0.19120 (11)	0.0404 (5)	
N6	0.84095 (18)	0.06202 (14)	0.35782 (13)	0.0667 (7)	
N7	0.47364 (12)	0.02113 (9)	0.12412 (9)	0.0322 (4)	
N8	0.35591 (12)	0.08611 (9)	0.18803 (9)	0.0317 (4)	
N9	0.46279 (13)	0.17553 (10)	0.25531 (10)	0.0398 (5)	
N10	0.19813 (18)	0.14241 (16)	0.35251 (14)	0.0758 (8)	
N11	0.16297 (13)	0.06318 (10)	0.19101 (11)	0.0406 (5)	
N12	0.15863 (15)	−0.01352 (12)	0.02869 (13)	0.0546 (6)	
C1	0.63266 (16)	0.02160 (12)	0.27350 (12)	0.0378 (5)	
C2	0.67286 (19)	−0.03481 (14)	0.31173 (15)	0.0552 (7)	
H2	0.7393	−0.0424	0.3162	0.066*	
C3	0.6137 (2)	−0.08021 (18)	0.34353 (18)	0.0745 (10)	
H3	0.6399	−0.1185	0.3698	0.089*	
C4	0.5165 (2)	−0.06812 (19)	0.33594 (18)	0.0758 (10)	
H4	0.4759	−0.0975	0.358	0.091*	
C5	0.4789 (2)	−0.01276 (15)	0.29575 (14)	0.0541 (7)	
H5	0.4123	−0.0054	0.2906	0.065*	
C6	0.57556 (16)	0.20229 (11)	0.11055 (12)	0.0352 (5)	
C7	0.58291 (18)	0.26305 (13)	0.07218 (13)	0.0442 (6)	
H7	0.643	0.2841	0.0705	0.053*	
C8	0.5013 (2)	0.29245 (14)	0.03640 (15)	0.0550 (7)	
H8	0.5055	0.3331	0.0096	0.066*	
C9	0.4138 (2)	0.26139 (15)	0.04053 (15)	0.0555 (7)	
H9	0.3581	0.2795	0.0151	0.067*	
C10	0.40864 (17)	0.20356 (13)	0.08221 (14)	0.0452 (6)	
H10	0.3483	0.184	0.0862	0.054*	
C11	0.65565 (15)	0.16187 (11)	0.14924 (11)	0.0328 (5)	
C12	0.75492 (16)	0.16547 (12)	0.14524 (13)	0.0386 (5)	
C13	0.68469 (16)	0.07427 (12)	0.23633 (11)	0.0343 (5)	
C14	0.78403 (16)	0.08768 (12)	0.23868 (12)	0.0373 (5)	
C15	0.85864 (17)	0.05717 (13)	0.29152 (15)	0.0462 (6)	
C16	0.94226 (18)	0.02824 (15)	0.27169 (17)	0.0570 (7)	
H16	0.9518	0.0263	0.2245	0.068*	
C17	1.0109 (2)	0.00245 (19)	0.3226 (2)	0.0787 (10)	
H17	1.0676	−0.0177	0.3107	0.094*	
C18	0.9946 (3)	0.0070 (2)	0.3902 (2)	0.0938 (12)	
H18	1.0405	−0.0098	0.4258	0.113*	
C19	0.9091 (3)	0.0368 (2)	0.4069 (2)	0.0945 (12)	
H19	0.8987	0.0392	0.4539	0.113*	
C20	0.79680 (17)	0.20729 (13)	0.09082 (16)	0.0461 (6)	
C21	0.8696 (2)	0.25517 (16)	0.1084 (2)	0.0729 (10)	
H21	0.8979	0.2607	0.1547	0.088*	
C22	0.8994 (3)	0.29532 (19)	0.0535 (3)	0.0974 (14)	
H22	0.9475	0.3293	0.0626	0.117*	
C23	0.8570 (3)	0.2838 (2)	−0.0131 (3)	0.0968 (14)	
H23	0.8752	0.3106	−0.05	0.116*	
C24	0.7888 (3)	0.23394 (19)	−0.02569 (19)	0.0808 (11)	
H24	0.7627	0.2259	−0.0721	0.097*	
C25	0.38166 (15)	−0.00599 (11)	0.11185 (11)	0.0322 (5)	
C26	0.36364 (17)	−0.06764 (12)	0.07343 (12)	0.0397 (5)	
H26	0.3016	−0.0866	0.0664	0.048*	
C27	0.43881 (18)	−0.10062 (12)	0.04572 (12)	0.0415 (6)	
H27	0.4276	−0.1418	0.0194	0.05*	
C28	0.53014 (18)	−0.07250 (13)	0.05702 (12)	0.0412 (6)	
H28	0.5813	−0.0941	0.0383	0.049*	
C29	0.54478 (16)	−0.01232 (12)	0.09616 (12)	0.0368 (5)	
H29	0.607	0.0064	0.1038	0.044*	
C30	0.36729 (16)	0.18459 (13)	0.26188 (12)	0.0405 (6)	
C31	0.33384 (19)	0.24506 (14)	0.29113 (16)	0.0572 (8)	
H31	0.2681	0.2511	0.2939	0.069*	
C32	0.3992 (2)	0.29638 (17)	0.31615 (19)	0.0777 (10)	
H32	0.3782	0.338	0.3351	0.093*	
C33	0.4960 (2)	0.28530 (17)	0.31276 (19)	0.0791 (11)	
H33	0.5414	0.3184	0.3313	0.095*	
C34	0.52507 (19)	0.22493 (15)	0.28178 (15)	0.0583 (8)	
H34	0.5907	0.2182	0.2791	0.07*	
C35	0.30654 (16)	0.12729 (12)	0.22915 (12)	0.0365 (5)	
C36	0.20994 (16)	0.11077 (13)	0.23360 (13)	0.0408 (6)	
C37	0.31129 (15)	0.03477 (11)	0.14734 (11)	0.0309 (5)	
C38	0.21093 (15)	0.02652 (12)	0.14645 (12)	0.0363 (5)	
C39	0.14880 (16)	−0.01974 (13)	0.09692 (15)	0.0441 (6)	
C40	0.08168 (19)	−0.06434 (15)	0.12202 (18)	0.0618 (8)	
H40	0.076	−0.0675	0.1698	0.074*	
C41	0.0233 (2)	−0.10417 (17)	0.0727 (3)	0.0876 (13)	
H41	−0.0227	−0.1347	0.0873	0.105*	
C42	0.0332 (3)	−0.09853 (19)	0.0033 (3)	0.0939 (15)	
H42	−0.0054	−0.125	−0.03	0.113*	
C43	0.1008 (2)	−0.05331 (18)	−0.01650 (18)	0.0735 (10)	
H43	0.1073	−0.0498	−0.0642	0.088*	
C44	0.15457 (18)	0.14415 (14)	0.28708 (16)	0.0531 (7)	
C45	0.06517 (19)	0.17234 (16)	0.26806 (19)	0.0681 (9)	
H45	0.036	0.1708	0.2217	0.082*	
C46	0.0191 (3)	0.20364 (19)	0.3210 (3)	0.0977 (14)	
H46	−0.0417	0.2242	0.3107	0.117*	
C47	0.0648 (3)	0.2036 (2)	0.3879 (3)	0.1118 (17)	
H47	0.036	0.2247	0.4239	0.134*	
C48	0.1513 (3)	0.1729 (2)	0.4015 (2)	0.1036 (15)	
H48	0.1809	0.1728	0.4477	0.124*	
N13	0.1612 (3)	0.2152 (2)	0.0631 (2)	0.1122 (13)	
C49	0.1565 (3)	0.2608 (3)	0.0965 (3)	0.0937 (12)	
N14	0.1473 (5)	0.3237 (3)	0.1229 (3)	0.186 (2)	
C50	0.1319 (4)	0.3337 (3)	0.1872 (4)	0.1124 (17)	
N15	0.1194 (4)	0.3529 (2)	0.2376 (3)	0.160 (2)	
O1W	0.24526 (17)	0.38984 (15)	0.48172 (14)	0.0834 (7)	
H11W	0.209 (2)	0.3545 (12)	0.4841 (17)	0.125*	
H12W	0.215 (2)	0.4268 (11)	0.493 (2)	0.125*	
O2W	0.2300 (2)	0.43951 (17)	0.34384 (15)	0.1059 (9)	
H21W	0.1789 (18)	0.423 (2)	0.3212 (18)	0.159*	
H22W	0.239 (3)	0.415 (2)	0.3818 (15)	0.159*	
O3W	0.3747 (7)	0.0893 (3)	0.4321 (5)	0.319 (5)	
H31W	0.331 (9)	0.076 (7)	0.399 (6)	0.478*	
H32W	0.385 (13)	0.053 (5)	0.459 (7)	0.478*	
O4W	0.537 (2)	0.0446 (14)	0.4763 (18)	0.69 (4)	0.5
H41W	0.597 (4)	0.041 (8)	0.474 (9)	1.035*	0.5
H42W	0.528 (12)	0.041 (13)	0.519 (4)	1.035*	0.5
O5WA	0.730 (2)	0.0261 (11)	0.5157 (8)	0.355 (15)	0.386 (8)
H51A	0.76 (3)	−0.004 (17)	0.494 (5)	0.533*	0.386 (8)
H52A	0.742 (12)	0.016 (3)	0.5594 (10)	0.533*	0.386 (8)
N16A	0.7525 (14)	0.0627 (10)	0.6648 (9)	0.126 (6)	0.386 (8)
C51A	0.7388 (13)	0.0951 (10)	0.7131 (10)	0.086 (5)	0.386 (8)
N17A	0.7190 (9)	0.1288 (7)	0.7671 (6)	0.105 (3)	0.386 (8)
C52A	0.7437 (18)	0.1931 (8)	0.7891 (9)	0.074 (4)	0.386 (8)
N18A	0.7701 (12)	0.2453 (7)	0.8021 (7)	0.082 (3)	0.386 (8)
O5WB	0.6583 (5)	0.1099 (6)	0.4317 (5)	0.220 (6)	0.614 (8)
H51B	0.697 (7)	0.119 (9)	0.402 (5)	0.33*	0.614 (8)
H52B	0.688 (9)	0.095 (8)	0.471 (3)	0.33*	0.614 (8)
N16B	0.7312 (13)	0.0732 (9)	0.5845 (8)	0.322 (11)	0.614 (8)
C51B	0.7422 (10)	0.0856 (7)	0.6450 (9)	0.163 (8)	0.614 (8)
N17B	0.7542 (14)	0.0954 (11)	0.7138 (11)	0.210 (9)	0.614 (8)
C52B	0.7444 (19)	0.1589 (11)	0.7443 (12)	0.205 (9)	0.614 (8)
N18B	0.7537 (16)	0.2094 (10)	0.7730 (8)	0.159 (7)	0.614 (8)

### Atomic displacement parameters (Å^2^)

**Table:**

	*U*^11^	*U*^22^	*U*^33^	*U*^12^	*U*^13^	*U*^23^
Fe1	0.02523 (16)	0.03431 (18)	0.03856 (19)	−0.00080 (13)	0.00675 (13)	−0.00539 (14)
N1	0.0337 (10)	0.0442 (11)	0.0374 (11)	−0.0032 (9)	0.0076 (9)	−0.0028 (9)
N2	0.0271 (9)	0.0333 (10)	0.0351 (10)	0.0012 (7)	0.0067 (8)	−0.0049 (8)
N3	0.0320 (10)	0.0313 (10)	0.0421 (11)	0.0021 (8)	0.0062 (8)	−0.0066 (8)
N4	0.0659 (15)	0.0559 (15)	0.0594 (16)	0.0089 (12)	0.0279 (13)	0.0078 (12)
N5	0.0292 (10)	0.0401 (11)	0.0524 (13)	−0.0029 (9)	0.0074 (9)	−0.0009 (9)
N6	0.0649 (16)	0.0805 (18)	0.0514 (16)	0.0088 (14)	−0.0064 (13)	−0.0002 (13)
N7	0.0303 (9)	0.0324 (10)	0.0342 (10)	0.0029 (8)	0.0048 (8)	−0.0006 (8)
N8	0.0254 (9)	0.0338 (10)	0.0360 (10)	0.0003 (7)	0.0042 (8)	−0.0050 (8)
N9	0.0318 (10)	0.0427 (11)	0.0460 (12)	−0.0060 (9)	0.0098 (9)	−0.0132 (9)
N10	0.0585 (16)	0.106 (2)	0.0671 (18)	−0.0184 (15)	0.0251 (14)	−0.0346 (16)
N11	0.0270 (10)	0.0425 (11)	0.0534 (13)	−0.0033 (8)	0.0088 (9)	−0.0102 (10)
N12	0.0415 (12)	0.0561 (14)	0.0617 (15)	0.0102 (10)	−0.0114 (11)	−0.0222 (11)
C1	0.0333 (12)	0.0415 (13)	0.0393 (13)	−0.0016 (10)	0.0075 (10)	−0.0012 (10)
C2	0.0409 (14)	0.0550 (17)	0.0700 (19)	0.0028 (13)	0.0079 (14)	0.0167 (14)
C3	0.0590 (19)	0.070 (2)	0.094 (3)	0.0021 (16)	0.0065 (18)	0.0389 (19)
C4	0.059 (2)	0.081 (2)	0.089 (2)	−0.0075 (17)	0.0181 (17)	0.040 (2)
C5	0.0408 (14)	0.0639 (18)	0.0591 (17)	−0.0052 (13)	0.0126 (13)	0.0122 (14)
C6	0.0360 (12)	0.0312 (12)	0.0401 (13)	−0.0006 (10)	0.0115 (10)	−0.0073 (10)
C7	0.0462 (14)	0.0341 (13)	0.0541 (16)	0.0003 (11)	0.0136 (12)	−0.0039 (11)
C8	0.0639 (19)	0.0403 (15)	0.0610 (18)	0.0089 (13)	0.0080 (15)	0.0037 (12)
C9	0.0487 (16)	0.0537 (17)	0.0621 (18)	0.0127 (13)	−0.0018 (14)	0.0050 (14)
C10	0.0336 (13)	0.0429 (14)	0.0583 (16)	0.0063 (11)	0.0024 (12)	−0.0041 (12)
C11	0.0312 (11)	0.0311 (11)	0.0368 (12)	−0.0018 (9)	0.0070 (10)	−0.0057 (9)
C12	0.0339 (12)	0.0339 (12)	0.0498 (14)	−0.0026 (10)	0.0122 (11)	−0.0054 (10)
C13	0.0311 (11)	0.0373 (12)	0.0348 (12)	0.0001 (10)	0.0054 (10)	−0.0050 (10)
C14	0.0304 (11)	0.0392 (13)	0.0419 (13)	−0.0001 (10)	0.0033 (10)	−0.0070 (10)
C15	0.0334 (13)	0.0451 (15)	0.0581 (17)	−0.0039 (11)	−0.0030 (12)	0.0035 (12)
C16	0.0349 (14)	0.0622 (18)	0.073 (2)	0.0034 (13)	0.0011 (13)	0.0119 (15)
C17	0.0435 (17)	0.077 (2)	0.112 (3)	0.0083 (15)	−0.0032 (19)	0.016 (2)
C18	0.076 (3)	0.096 (3)	0.099 (3)	0.007 (2)	−0.031 (2)	0.021 (2)
C19	0.108 (3)	0.108 (3)	0.061 (2)	0.011 (3)	−0.017 (2)	0.008 (2)
C20	0.0326 (12)	0.0365 (13)	0.0729 (19)	0.0045 (10)	0.0211 (13)	0.0083 (12)
C21	0.0394 (15)	0.0529 (18)	0.129 (3)	−0.0093 (13)	0.0207 (17)	0.0132 (18)
C22	0.059 (2)	0.061 (2)	0.179 (5)	−0.0118 (17)	0.041 (3)	0.029 (3)
C23	0.085 (3)	0.079 (3)	0.140 (4)	0.015 (2)	0.068 (3)	0.045 (3)
C24	0.089 (2)	0.080 (2)	0.084 (2)	0.019 (2)	0.051 (2)	0.0264 (19)
C25	0.0303 (11)	0.0317 (12)	0.0344 (12)	0.0027 (9)	0.0030 (9)	0.0003 (9)
C26	0.0369 (13)	0.0345 (12)	0.0465 (14)	0.0022 (10)	−0.0002 (11)	−0.0053 (11)
C27	0.0494 (14)	0.0351 (12)	0.0397 (13)	0.0086 (11)	0.0038 (11)	−0.0053 (10)
C28	0.0450 (14)	0.0427 (13)	0.0374 (13)	0.0147 (11)	0.0116 (11)	0.0007 (11)
C29	0.0328 (12)	0.0413 (13)	0.0376 (13)	0.0066 (10)	0.0092 (10)	0.0010 (10)
C30	0.0311 (12)	0.0467 (14)	0.0451 (14)	−0.0039 (10)	0.0105 (10)	−0.0126 (11)
C31	0.0414 (14)	0.0531 (17)	0.080 (2)	−0.0011 (12)	0.0172 (14)	−0.0296 (15)
C32	0.0595 (19)	0.067 (2)	0.110 (3)	−0.0119 (16)	0.0254 (19)	−0.0512 (19)
C33	0.0541 (18)	0.074 (2)	0.113 (3)	−0.0228 (16)	0.0237 (18)	−0.058 (2)
C34	0.0362 (14)	0.0693 (19)	0.0705 (19)	−0.0126 (13)	0.0104 (13)	−0.0332 (16)
C35	0.0306 (11)	0.0365 (12)	0.0431 (13)	−0.0004 (10)	0.0072 (10)	−0.0091 (10)
C36	0.0282 (11)	0.0453 (14)	0.0504 (15)	−0.0007 (10)	0.0105 (11)	−0.0108 (11)
C37	0.0289 (11)	0.0290 (11)	0.0344 (12)	−0.0001 (9)	0.0017 (9)	−0.0025 (9)
C38	0.0312 (11)	0.0331 (12)	0.0445 (14)	−0.0015 (9)	0.0040 (10)	−0.0045 (10)
C39	0.0254 (11)	0.0392 (13)	0.0659 (18)	0.0029 (10)	−0.0011 (11)	−0.0165 (12)
C40	0.0383 (14)	0.0463 (16)	0.100 (2)	−0.0054 (12)	0.0051 (15)	−0.0142 (15)
C41	0.0416 (17)	0.0523 (19)	0.165 (4)	−0.0093 (14)	−0.005 (2)	−0.026 (2)
C42	0.053 (2)	0.070 (2)	0.146 (4)	0.0201 (18)	−0.036 (2)	−0.060 (3)
C43	0.0559 (19)	0.078 (2)	0.078 (2)	0.0301 (18)	−0.0244 (17)	−0.0407 (18)
C44	0.0373 (14)	0.0535 (16)	0.073 (2)	−0.0141 (12)	0.0237 (14)	−0.0243 (14)
C45	0.0397 (15)	0.0616 (19)	0.108 (3)	−0.0053 (14)	0.0306 (16)	−0.0263 (18)
C46	0.051 (2)	0.074 (2)	0.179 (4)	−0.0058 (17)	0.058 (3)	−0.040 (3)
C47	0.087 (3)	0.125 (4)	0.137 (4)	−0.035 (3)	0.071 (3)	−0.073 (3)
C48	0.086 (3)	0.146 (4)	0.087 (3)	−0.039 (3)	0.047 (2)	−0.055 (3)
N13	0.080 (2)	0.111 (3)	0.148 (4)	0.006 (2)	0.028 (2)	−0.020 (3)
C49	0.072 (2)	0.088 (3)	0.122 (4)	−0.007 (2)	0.017 (2)	−0.003 (3)
N14	0.276 (7)	0.111 (4)	0.177 (5)	−0.039 (4)	0.051 (5)	−0.022 (4)
C50	0.118 (4)	0.081 (3)	0.131 (4)	−0.017 (3)	−0.014 (4)	−0.026 (3)
N15	0.247 (6)	0.085 (3)	0.139 (4)	0.021 (3)	−0.008 (4)	−0.016 (3)
O1W	0.0685 (15)	0.1015 (19)	0.0809 (17)	0.0102 (13)	0.0121 (13)	0.0123 (15)
O2W	0.106 (2)	0.127 (2)	0.0831 (19)	−0.0274 (18)	0.0043 (16)	0.0215 (16)
O3W	0.342 (9)	0.207 (6)	0.350 (10)	0.050 (6)	−0.194 (7)	−0.082 (6)
O4W	0.61 (5)	0.69 (5)	0.67 (4)	0.42 (5)	−0.34 (4)	−0.59 (5)
O5WA	0.68 (5)	0.227 (19)	0.190 (16)	0.04 (2)	0.19 (2)	−0.041 (14)
N16A	0.058 (7)	0.113 (13)	0.205 (15)	0.010 (8)	0.013 (10)	−0.033 (11)
C51A	0.044 (6)	0.060 (8)	0.154 (14)	−0.017 (6)	0.007 (8)	0.015 (9)
N17A	0.080 (7)	0.113 (7)	0.128 (9)	−0.014 (6)	0.034 (6)	0.037 (6)
C52A	0.061 (8)	0.104 (7)	0.061 (10)	0.009 (6)	0.017 (7)	0.039 (6)
N18A	0.077 (8)	0.109 (7)	0.058 (7)	0.001 (5)	−0.001 (6)	0.012 (5)
O5WB	0.144 (6)	0.251 (11)	0.272 (11)	−0.049 (6)	0.056 (6)	−0.174 (9)
N16B	0.43 (2)	0.195 (13)	0.36 (2)	−0.024 (14)	0.104 (19)	0.050 (17)
C51B	0.071 (7)	0.106 (8)	0.31 (2)	0.004 (6)	−0.001 (11)	0.124 (11)
N17B	0.117 (11)	0.200 (17)	0.310 (19)	0.009 (13)	0.016 (14)	0.156 (15)
C52B	0.140 (13)	0.28 (2)	0.21 (2)	0.009 (19)	0.055 (14)	0.122 (14)
N18B	0.063 (7)	0.33 (2)	0.084 (11)	0.020 (14)	0.020 (8)	0.101 (11)

### Geometric parameters (Å, °)

**Table:**

Fe1—N8	1.8719 (17)	C23—H23	0.93
Fe1—N2	1.8737 (17)	C24—H24	0.93
Fe1—N7	1.9557 (18)	C25—C26	1.386 (3)
Fe1—N9	1.9573 (18)	C25—C37	1.476 (3)
Fe1—N1	1.9594 (19)	C26—C27	1.379 (3)
Fe1—N3	1.9653 (19)	C26—H26	0.93
N1—C5	1.338 (3)	C27—C28	1.371 (3)
N1—C1	1.370 (3)	C27—H27	0.93
N2—C13	1.345 (3)	C28—C29	1.365 (3)
N2—C11	1.352 (3)	C28—H28	0.93
N3—C10	1.341 (3)	C29—H29	0.93
N3—C6	1.364 (3)	C30—C31	1.380 (3)
N4—C24	1.332 (4)	C30—C35	1.468 (3)
N4—C20	1.338 (3)	C31—C32	1.377 (4)
N5—C14	1.339 (3)	C31—H31	0.93
N5—C12	1.340 (3)	C32—C33	1.372 (4)
N6—C15	1.325 (3)	C32—H32	0.93
N6—C19	1.339 (4)	C33—C34	1.371 (4)
N7—C29	1.341 (3)	C33—H33	0.93
N7—C25	1.373 (3)	C34—H34	0.93
N8—C37	1.348 (3)	C35—C36	1.393 (3)
N8—C35	1.353 (3)	C36—C44	1.495 (3)
N9—C34	1.332 (3)	C37—C38	1.404 (3)
N9—C30	1.361 (3)	C38—C39	1.488 (3)
N10—C44	1.324 (4)	C39—C40	1.388 (4)
N10—C48	1.336 (4)	C40—C41	1.390 (4)
N11—C36	1.332 (3)	C40—H40	0.93
N11—C38	1.339 (3)	C41—C42	1.357 (6)
N12—C39	1.335 (3)	C41—H41	0.93
N12—C43	1.339 (4)	C42—C43	1.360 (5)
C1—C2	1.374 (3)	C42—H42	0.93
C1—C13	1.466 (3)	C43—H43	0.93
C2—C3	1.382 (4)	C44—C45	1.362 (4)
C2—H2	0.93	C45—C46	1.396 (5)
C3—C4	1.363 (4)	C45—H45	0.93
C3—H3	0.93	C46—C47	1.358 (6)
C4—C5	1.366 (4)	C46—H46	0.93
C4—H4	0.93	C47—C48	1.334 (6)
C5—H5	0.93	C47—H47	0.93
C6—C7	1.375 (3)	C48—H48	0.93
C6—C11	1.475 (3)	N13—C49	1.082 (5)
C7—C8	1.371 (4)	C49—N14	1.305 (6)
C7—H7	0.93	N14—C50	1.288 (7)
C8—C9	1.363 (4)	C50—N15	1.066 (6)
C8—H8	0.93	O1W—H11W	0.84 (2)
C9—C10	1.362 (4)	O1W—H12W	0.86 (2)
C9—H9	0.93	O2W—H21W	0.85 (3)
C10—H10	0.93	O2W—H22W	0.86 (3)
C11—C12	1.395 (3)	O3W—H31W	0.86 (11)
C12—C20	1.483 (3)	O3W—H32W	0.86 (12)
C13—C14	1.401 (3)	O4W—H41W	0.84 (7)
C14—C15	1.476 (3)	O4W—H42W	0.85 (9)
C15—C16	1.381 (4)	O5WA—H51A	0.9 (3)
C16—C17	1.369 (4)	O5WA—H52A	0.85 (4)
C16—H16	0.93	O5WB—H51B	0.85 (11)
C17—C18	1.344 (5)	O5WB—H52B	0.86 (8)
C17—H17	0.93	N16A—C51A	1.145 (14)
C18—C19	1.390 (5)	C51A—N17A	1.273 (14)
C18—H18	0.93	N17A—C52A	1.321 (15)
C19—H19	0.93	C52A—N18A	1.072 (15)
C20—C21	1.371 (4)	N16B—C51B	1.173 (16)
C21—C22	1.400 (5)	N16B—H52A	1.19 (5)
C21—H21	0.93	C51B—N17B	1.320 (16)
C22—C23	1.355 (6)	N17B—C52B	1.351 (16)
C22—H22	0.93	C52B—N18B	1.101 (17)
C23—C24	1.339 (6)		
			
N8—Fe1—N2	179.59 (8)	C20—C21—H21	121.6
N8—Fe1—N7	81.02 (7)	C22—C21—H21	121.6
N2—Fe1—N7	98.67 (7)	C23—C22—C21	118.9 (4)
N8—Fe1—N9	81.63 (7)	C23—C22—H22	120.5
N2—Fe1—N9	98.68 (7)	C21—C22—H22	120.5
N7—Fe1—N9	162.64 (7)	C24—C23—C22	120.0 (4)
N8—Fe1—N1	98.78 (8)	C24—C23—H23	120
N2—Fe1—N1	80.92 (8)	C22—C23—H23	120
N7—Fe1—N1	87.40 (8)	N4—C24—C23	123.4 (4)
N9—Fe1—N1	95.77 (8)	N4—C24—H24	118.3
N8—Fe1—N3	98.93 (8)	C23—C24—H24	118.3
N2—Fe1—N3	81.37 (8)	N7—C25—C26	120.7 (2)
N7—Fe1—N3	95.31 (7)	N7—C25—C37	112.54 (18)
N9—Fe1—N3	86.86 (8)	C26—C25—C37	126.7 (2)
N1—Fe1—N3	162.29 (8)	C27—C26—C25	119.2 (2)
C5—N1—C1	118.4 (2)	C27—C26—H26	120.4
C5—N1—Fe1	126.28 (17)	C25—C26—H26	120.4
C1—N1—Fe1	114.56 (15)	C28—C27—C26	119.8 (2)
C13—N2—C11	121.29 (18)	C28—C27—H27	120.1
C13—N2—Fe1	119.79 (15)	C26—C27—H27	120.1
C11—N2—Fe1	118.91 (14)	C29—C28—C27	118.9 (2)
C10—N3—C6	118.1 (2)	C29—C28—H28	120.5
C10—N3—Fe1	126.68 (17)	C27—C28—H28	120.5
C6—N3—Fe1	114.63 (15)	N7—C29—C28	123.0 (2)
C24—N4—C20	116.9 (3)	N7—C29—H29	118.5
C14—N5—C12	119.50 (18)	C28—C29—H29	118.5
C15—N6—C19	116.6 (3)	N9—C30—C31	121.6 (2)
C29—N7—C25	118.31 (19)	N9—C30—C35	112.69 (19)
C29—N7—Fe1	126.17 (15)	C31—C30—C35	125.5 (2)
C25—N7—Fe1	115.15 (14)	C32—C31—C30	119.0 (2)
C37—N8—C35	121.34 (18)	C32—C31—H31	120.5
C37—N8—Fe1	120.12 (14)	C30—C31—H31	120.5
C35—N8—Fe1	118.52 (14)	C33—C32—C31	119.1 (3)
C34—N9—C30	118.3 (2)	C33—C32—H32	120.4
C34—N9—Fe1	126.04 (16)	C31—C32—H32	120.4
C30—N9—Fe1	114.39 (15)	C34—C33—C32	119.4 (3)
C44—N10—C48	116.5 (3)	C34—C33—H33	120.3
C36—N11—C38	119.65 (18)	C32—C33—H33	120.3
C39—N12—C43	117.2 (3)	N9—C34—C33	122.4 (2)
N1—C1—C2	120.8 (2)	N9—C34—H34	118.8
N1—C1—C13	112.7 (2)	C33—C34—H34	118.8
C2—C1—C13	126.4 (2)	N8—C35—C36	118.0 (2)
C1—C2—C3	119.5 (3)	N8—C35—C30	111.52 (19)
C1—C2—H2	120.3	C36—C35—C30	130.5 (2)
C3—C2—H2	120.3	N11—C36—C35	121.3 (2)
C4—C3—C2	119.1 (3)	N11—C36—C44	116.7 (2)
C4—C3—H3	120.5	C35—C36—C44	122.0 (2)
C2—C3—H3	120.5	N8—C37—C38	118.34 (19)
C3—C4—C5	119.7 (3)	N8—C37—C25	110.96 (18)
C3—C4—H4	120.1	C38—C37—C25	130.67 (19)
C5—C4—H4	120.1	N11—C38—C37	120.5 (2)
N1—C5—C4	122.3 (3)	N11—C38—C39	114.50 (19)
N1—C5—H5	118.8	C37—C38—C39	125.0 (2)
C4—C5—H5	118.8	N12—C39—C40	123.1 (2)
N3—C6—C7	120.8 (2)	N12—C39—C38	116.8 (2)
N3—C6—C11	112.25 (19)	C40—C39—C38	120.1 (3)
C7—C6—C11	126.9 (2)	C39—C40—C41	117.2 (3)
C8—C7—C6	119.7 (2)	C39—C40—H40	121.4
C8—C7—H7	120.2	C41—C40—H40	121.4
C6—C7—H7	120.2	C42—C41—C40	120.0 (3)
C9—C8—C7	119.2 (3)	C42—C41—H41	120
C9—C8—H8	120.4	C40—C41—H41	120
C7—C8—H8	120.4	C41—C42—C43	118.7 (3)
C10—C9—C8	119.5 (3)	C41—C42—H42	120.7
C10—C9—H9	120.2	C43—C42—H42	120.7
C8—C9—H9	120.2	N12—C43—C42	123.8 (4)
N3—C10—C9	122.5 (2)	N12—C43—H43	118.1
N3—C10—H10	118.8	C42—C43—H43	118.1
C9—C10—H10	118.8	N10—C44—C45	124.1 (3)
N2—C11—C12	118.4 (2)	N10—C44—C36	115.0 (2)
N2—C11—C6	111.74 (18)	C45—C44—C36	120.9 (3)
C12—C11—C6	129.8 (2)	C44—C45—C46	117.3 (3)
N5—C12—C11	120.6 (2)	C44—C45—H45	121.4
N5—C12—C20	116.60 (19)	C46—C45—H45	121.4
C11—C12—C20	122.8 (2)	C47—C46—C45	118.7 (4)
N2—C13—C14	118.2 (2)	C47—C46—H46	120.6
N2—C13—C1	111.17 (19)	C45—C46—H46	120.6
C14—C13—C1	130.7 (2)	C48—C47—C46	119.5 (4)
N5—C14—C13	120.8 (2)	C48—C47—H47	120.3
N5—C14—C15	115.2 (2)	C46—C47—H47	120.3
C13—C14—C15	124.1 (2)	C47—C48—N10	123.9 (4)
N6—C15—C16	123.5 (2)	C47—C48—H48	118.1
N6—C15—C14	115.6 (2)	N10—C48—H48	118.1
C16—C15—C14	120.9 (2)	N13—C49—N14	166.7 (6)
C17—C16—C15	119.0 (3)	C50—N14—C49	122.7 (5)
C17—C16—H16	120.5	N15—C50—N14	168.6 (6)
C15—C16—H16	120.5	H11W—O1W—H12W	109 (3)
C18—C17—C16	118.5 (3)	H21W—O2W—H22W	105 (4)
C18—C17—H17	120.7	H31W—O3W—H32W	105 (13)
C16—C17—H17	120.7	H41W—O4W—H42W	108 (17)
C17—C18—C19	119.8 (3)	H51A—O5WA—H52A	105 (13)
C17—C18—H18	120.1	H51B—O5WB—H52B	113 (10)
C19—C18—H18	120.1	N16A—C51A—N17A	176.5 (17)
N6—C19—C18	122.5 (4)	C51A—N17A—C52A	130.4 (13)
N6—C19—H19	118.7	N18A—C52A—N17A	172.7 (18)
C18—C19—H19	118.7	C51B—N16B—H52A	125 (2)
N4—C20—C21	123.7 (3)	N16B—C51B—N17B	176.5 (17)
N4—C20—C12	114.7 (2)	C51B—N17B—C52B	123.3 (19)
C21—C20—C12	121.5 (3)	N18B—C52B—N17B	167 (3)
C20—C21—C22	116.9 (4)		
			
N8—Fe1—N1—C5	3.0 (2)	N2—C13—C14—C15	169.9 (2)
N2—Fe1—N1—C5	−176.7 (2)	C1—C13—C14—C15	−11.6 (4)
N7—Fe1—N1—C5	−77.5 (2)	C19—N6—C15—C16	−0.3 (4)
N9—Fe1—N1—C5	85.4 (2)	C19—N6—C15—C14	−177.9 (3)
N3—Fe1—N1—C5	−176.8 (2)	N5—C14—C15—N6	132.0 (2)
N8—Fe1—N1—C1	172.95 (15)	C13—C14—C15—N6	−48.3 (3)
N2—Fe1—N1—C1	−6.76 (15)	N5—C14—C15—C16	−45.7 (3)
N7—Fe1—N1—C1	92.46 (16)	C13—C14—C15—C16	134.0 (3)
N9—Fe1—N1—C1	−104.65 (16)	N6—C15—C16—C17	0.5 (4)
N3—Fe1—N1—C1	−6.9 (3)	C14—C15—C16—C17	178.0 (3)
N7—Fe1—N2—C13	−84.51 (17)	C15—C16—C17—C18	−0.7 (5)
N9—Fe1—N2—C13	95.93 (17)	C16—C17—C18—C19	0.6 (6)
N1—Fe1—N2—C13	1.42 (16)	C15—N6—C19—C18	0.2 (5)
N3—Fe1—N2—C13	−178.62 (17)	C17—C18—C19—N6	−0.4 (6)
N7—Fe1—N2—C11	94.33 (16)	C24—N4—C20—C21	−1.7 (4)
N9—Fe1—N2—C11	−85.23 (16)	C24—N4—C20—C12	177.0 (2)
N1—Fe1—N2—C11	−179.73 (16)	N5—C12—C20—N4	129.0 (2)
N3—Fe1—N2—C11	0.22 (15)	C11—C12—C20—N4	−50.5 (3)
N8—Fe1—N3—C10	2.4 (2)	N5—C12—C20—C21	−52.2 (3)
N2—Fe1—N3—C10	−177.9 (2)	C11—C12—C20—C21	128.3 (3)
N7—Fe1—N3—C10	84.10 (19)	N4—C20—C21—C22	2.9 (4)
N9—Fe1—N3—C10	−78.63 (19)	C12—C20—C21—C22	−175.7 (3)
N1—Fe1—N3—C10	−177.8 (2)	C20—C21—C22—C23	−1.4 (5)
N8—Fe1—N3—C6	173.59 (14)	C21—C22—C23—C24	−1.1 (6)
N2—Fe1—N3—C6	−6.70 (14)	C20—N4—C24—C23	−1.1 (5)
N7—Fe1—N3—C6	−104.70 (15)	C22—C23—C24—N4	2.5 (6)
N9—Fe1—N3—C6	92.57 (15)	C29—N7—C25—C26	2.0 (3)
N1—Fe1—N3—C6	−6.6 (3)	Fe1—N7—C25—C26	−171.45 (17)
N8—Fe1—N7—C29	−175.68 (19)	C29—N7—C25—C37	178.33 (18)
N2—Fe1—N7—C29	4.04 (19)	Fe1—N7—C25—C37	4.8 (2)
N9—Fe1—N7—C29	−177.4 (2)	N7—C25—C26—C27	−1.9 (3)
N1—Fe1—N7—C29	−76.36 (18)	C37—C25—C26—C27	−177.6 (2)
N3—Fe1—N7—C29	86.09 (18)	C25—C26—C27—C28	0.6 (4)
N8—Fe1—N7—C25	−2.77 (15)	C26—C27—C28—C29	0.4 (4)
N2—Fe1—N7—C25	176.95 (15)	C25—N7—C29—C28	−1.0 (3)
N9—Fe1—N7—C25	−4.5 (4)	Fe1—N7—C29—C28	171.73 (17)
N1—Fe1—N7—C25	96.54 (15)	C27—C28—C29—N7	−0.3 (4)
N3—Fe1—N7—C25	−101.00 (15)	C34—N9—C30—C31	4.0 (4)
N7—Fe1—N8—C37	−0.10 (16)	Fe1—N9—C30—C31	−163.9 (2)
N9—Fe1—N8—C37	179.37 (18)	C34—N9—C30—C35	178.7 (2)
N1—Fe1—N8—C37	−86.03 (17)	Fe1—N9—C30—C35	10.8 (3)
N3—Fe1—N8—C37	93.92 (17)	N9—C30—C31—C32	−2.0 (4)
N7—Fe1—N8—C35	178.32 (18)	C35—C30—C31—C32	−176.0 (3)
N9—Fe1—N8—C35	−2.21 (17)	C30—C31—C32—C33	−1.5 (5)
N1—Fe1—N8—C35	92.39 (17)	C31—C32—C33—C34	2.9 (6)
N3—Fe1—N8—C35	−87.66 (17)	C30—N9—C34—C33	−2.5 (4)
N8—Fe1—N9—C34	−172.0 (2)	Fe1—N9—C34—C33	163.8 (3)
N2—Fe1—N9—C34	8.3 (2)	C32—C33—C34—N9	−0.9 (6)
N7—Fe1—N9—C34	−170.2 (3)	C37—N8—C35—C36	6.1 (3)
N1—Fe1—N9—C34	90.0 (2)	Fe1—N8—C35—C36	−172.28 (17)
N3—Fe1—N9—C34	−72.4 (2)	C37—N8—C35—C30	−173.1 (2)
N8—Fe1—N9—C30	−5.17 (17)	Fe1—N8—C35—C30	8.5 (3)
N2—Fe1—N9—C30	175.11 (17)	N9—C30—C35—N8	−12.3 (3)
N7—Fe1—N9—C30	−3.4 (4)	C31—C30—C35—N8	162.2 (3)
N1—Fe1—N9—C30	−103.23 (17)	N9—C30—C35—C36	168.6 (2)
N3—Fe1—N9—C30	94.34 (18)	C31—C30—C35—C36	−16.9 (4)
C5—N1—C1—C2	4.5 (3)	C38—N11—C36—C35	5.0 (4)
Fe1—N1—C1—C2	−166.2 (2)	C38—N11—C36—C44	−173.7 (2)
C5—N1—C1—C13	−178.8 (2)	N8—C35—C36—N11	−10.0 (4)
Fe1—N1—C1—C13	10.4 (2)	C30—C35—C36—N11	169.1 (2)
N1—C1—C2—C3	−3.5 (4)	N8—C35—C36—C44	168.6 (2)
C13—C1—C2—C3	−179.7 (3)	C30—C35—C36—C44	−12.3 (4)
C1—C2—C3—C4	0.3 (5)	C35—N8—C37—C38	2.4 (3)
C2—C3—C4—C5	1.7 (6)	Fe1—N8—C37—C38	−179.26 (16)
C1—N1—C5—C4	−2.5 (4)	C35—N8—C37—C25	−175.71 (19)
Fe1—N1—C5—C4	167.1 (3)	Fe1—N8—C37—C25	2.7 (2)
C3—C4—C5—N1	−0.6 (5)	N7—C25—C37—N8	−4.7 (3)
C10—N3—C6—C7	4.8 (3)	C26—C25—C37—N8	171.3 (2)
Fe1—N3—C6—C7	−167.20 (17)	N7—C25—C37—C38	177.5 (2)
C10—N3—C6—C11	−176.75 (19)	C26—C25—C37—C38	−6.5 (4)
Fe1—N3—C6—C11	11.3 (2)	C36—N11—C38—C37	3.9 (4)
N3—C6—C7—C8	−4.9 (3)	C36—N11—C38—C39	−174.3 (2)
C11—C6—C7—C8	176.9 (2)	N8—C37—C38—N11	−7.6 (3)
C6—C7—C8—C9	1.1 (4)	C25—C37—C38—N11	170.0 (2)
C7—C8—C9—C10	2.6 (4)	N8—C37—C38—C39	170.4 (2)
C6—N3—C10—C9	−1.0 (3)	C25—C37—C38—C39	−12.0 (4)
Fe1—N3—C10—C9	169.9 (2)	C43—N12—C39—C40	−0.7 (4)
C8—C9—C10—N3	−2.7 (4)	C43—N12—C39—C38	−177.9 (2)
C13—N2—C11—C12	6.4 (3)	N11—C38—C39—N12	128.8 (2)
Fe1—N2—C11—C12	−172.42 (15)	C37—C38—C39—N12	−49.3 (3)
C13—N2—C11—C6	−175.49 (18)	N11—C38—C39—C40	−48.5 (3)
Fe1—N2—C11—C6	5.7 (2)	C37—C38—C39—C40	133.4 (3)
N3—C6—C11—N2	−10.8 (3)	N12—C39—C40—C41	0.4 (4)
C7—C6—C11—N2	167.5 (2)	C38—C39—C40—C41	177.5 (2)
N3—C6—C11—C12	167.0 (2)	C39—C40—C41—C42	0.0 (5)
C7—C6—C11—C12	−14.7 (4)	C40—C41—C42—C43	−0.2 (5)
C14—N5—C12—C11	3.9 (3)	C39—N12—C43—C42	0.6 (4)
C14—N5—C12—C20	−175.6 (2)	C41—C42—C43—N12	−0.1 (5)
N2—C11—C12—N5	−10.5 (3)	C48—N10—C44—C45	−2.6 (5)
C6—C11—C12—N5	171.8 (2)	C48—N10—C44—C36	178.8 (3)
N2—C11—C12—C20	169.1 (2)	N11—C36—C44—N10	128.7 (3)
C6—C11—C12—C20	−8.6 (4)	C35—C36—C44—N10	−50.0 (4)
C11—N2—C13—C14	3.7 (3)	N11—C36—C44—C45	−49.9 (4)
Fe1—N2—C13—C14	−177.50 (15)	C35—C36—C44—C45	131.4 (3)
C11—N2—C13—C1	−175.07 (18)	N10—C44—C45—C46	2.6 (5)
Fe1—N2—C13—C1	3.8 (2)	C36—C44—C45—C46	−178.9 (3)
N1—C1—C13—N2	−9.1 (3)	C44—C45—C46—C47	−0.8 (5)
C2—C1—C13—N2	167.4 (2)	C45—C46—C47—C48	−0.9 (6)
N1—C1—C13—C14	172.4 (2)	C46—C47—C48—N10	1.0 (7)
C2—C1—C13—C14	−11.2 (4)	C44—N10—C48—C47	0.7 (6)
C12—N5—C14—C13	6.6 (3)	N13—C49—N14—C50	164 (2)
C12—N5—C14—C15	−173.8 (2)	C49—N14—C50—N15	180 (10)
N2—C13—C14—N5	−10.5 (3)	C51B—N17B—C52B—N18B	−114 (12)
C1—C13—C14—N5	168.0 (2)		

### Hydrogen-bond geometry (Å, °)

**Table:**

*D*—H···*A*	*D*—H	H···*A*	*D*···*A*	*D*—H···*A*
O1W—H11W···N13^i^	0.84 (2)	2.17 (3)	2.862 (5)	139 (3)
O1W—H12W···N12^i^	0.86 (2)	1.98 (2)	2.830 (4)	173 (3)
O2W—H21W···N15	0.85 (3)	2.16 (4)	2.903 (6)	146 (3)
O2W—H22W···O1W	0.86 (3)	1.96 (3)	2.785 (4)	160 (4)
O3W—H31W···N10	0.86 (11)	2.32 (13)	2.909 (10)	126 (11)
O3W—H32W···O5WA^ii^	0.86 (12)	2.29 (15)	2.87 (2)	126 (15)
O3W—H32W···O4W	0.86 (12)	2.11 (18)	2.46 (3)	104 (13)
O3W—H32W···O4W^ii^	0.86 (12)	2.41 (12)	3.24 (3)	162 (15)
O4W—H41W···O5WA	0.85 (7)	1.95 (9)	2.72 (4)	153 (17)
O5WA—H52A···N16A	0.85 (4)	2.19 (4)	2.92 (2)	143 (7)
O5WB—H51B···N6	0.85 (11)	2.52 (12)	3.188 (8)	136 (13)
O5WB—H52B···N16B	0.86 (8)	2.22 (7)	3.057 (18)	164 (10)
C8—H8···O4W^iii^	0.93	2.45	3.349 (16)	162

Symmetry codes: (i) *x*, −*y*+1/2, *z*+1/2; (ii) −*x*+1, −*y*, −*z*+1; (iii) *x*, −*y*+1/2, *z*−1/2.
